# The Health Risk and Source Assessment of Polycyclic Aromatic Hydrocarbons (PAHs) in the Soil of Industrial Cities in India

**DOI:** 10.3390/toxics11060515

**Published:** 2023-06-08

**Authors:** Tapan Kumar Sankar, Amit Kumar, Dilip Kumar Mahto, Kailash Chandra Das, Prakash Narayan, Manish Fukate, Prashant Awachat, Dhanshri Padghan, Faruq Mohammad, Hamad A. Al-Lohedan, Ahmed A. Soleiman, Balram Ambade

**Affiliations:** 1 School of Science, Engineering and Technology, G H Raisoni University, Amravati 444701, India; 2 Department of Environmental Engineering and Management, Chaoyang University of Technology, Taichung 43149, Taiwan; 3 Department of Chemistry, National Institute of Technology, Jamshedpur 831014, India; 4 Department of Chemistry, College of Science, King Saud University, Riyadh 11451, Saudi Arabia; 5 College of Sciences and Engineering, Southern University and A&M College, Baton Rouge, LA 70813, USA

**Keywords:** diagnostics ratio, ILCR, PAHs, industrial area, potential risk

## Abstract

Industrial areas play an important role in the urban ecosystem. Industrial site environmental quality is linked to human health. Soil samples from two different cities in India, Jamshedpur and Amravati, were collected and analyzed to assess the sources of polycyclic aromatic hydrocarbons (PAHs) in industrial areas and their potential health risks. The total concentration of 16 PAHs in JSR (Jamshedpur) varied from 1662.90 to 10,879.20 ng/g, whereas the concentration ranged from 1456.22 to 5403.45 ng/g in the soil of AMT (Amravati). The PAHs in the samples were dominated by four-ring PAHs, followed by five-ring PAHs, and a small percentage of two-ring PAHs. The ILCR (incremental lifetime cancer risk) of the soil of Amravati was lower compared to that of Jamshedpur. The risk due to PAH exposure for children and adults was reported to be in the order of ingestion > dermal contact > inhalation while for adolescents it was dermal contact > ingestion > inhalation in Jamshedpur. In contrast, in the soil of Amravati, the PAH exposure path risk for children and adolescents were the same and showed the following order: dermal contact > ingestion > inhalation while for the adulthood age group, the order was ingestion > dermal contact > inhalation. The diagnostic ratio approach was used to assess the sources of PAHs in various environmental media. The PAH sources were mainly dominated by coal and petroleum/oil combustion. As both the study areas belong to industrial sites, the significant sources were industrial emissions, followed by traffic emissions, coal combustion for domestic livelihood, as well as due to the geographical location of the sampling sites. The results of this investigation provide novel information for contamination evaluation and human health risk assessment in PAH-contaminated sites in India.

## 1. Introduction

PAHs have become a global concern in the last few decades because of their carcinogenic, mutagenic, and teratogenic nature [1,2]. PAHs are a group of ubiquitous organic pollutants that are composed of two or more fused aromatic rings of composed of carbon and hydrogen [1,2,3,4]. The primary non-occupational sources of exposure to PAHs are combustion processes in motor cars, petroleum refineries, fossil fuel-burning power plants, coking plants, asphalt production facilities, metal foundries, burning of crop residue and grass, bush fires, smoking, and food preparation [5]. The bulk of hazardous PAHs is produced by coal emissions, tobacco smoke, and vehicle exhaust [6]. The emissions of motor vehicles and commercial transport are the main contributors to atmospheric PAHs [7,8,9,10,11,12].

The United States Environmental Protection Agency (USEPA) has listed 16 PAHs as having toxicity potential, since they are widely distributed in the natural environment, including in the air, water, and soils [13,14,15]. Among them, seven PAHs are classified as B2 class carcinogens namely benzo [a] anthracene (BaA), benzo [a] pyrene (BaP), benzo [b] fluoranthene (BbF), benzo [k] fluoranthene (BkF), chrysene (Chr), dibenzo [a,h] anthracene (DBahA), and indeno [1,2,3-c,d] pyrene (IcP) [16]. There are several routes of human and animal exposure to PAHs, such as dermal contact with tar and soot, direct contact with the soil, consumption of smoke-tainted foods and tainted water, and direct inhalation of cigarette smoke and polluted air [1]. A previous study reported that soil PAH exposure is a greater risk to human health than air and water PAHs [17]. The broad distribution of PAHs around the planet is a result of their long-range atmospheric transport (LRAT) and environmental persistence. In the presence of suitable climatic circumstances, PAHs can travel long distances before being precipitated onto land, water, and vegetation [18,19,20,21,22]. The top layer of soil contains a large portion of the combustion-derived PAHs [23]. It is anticipated that the majority of PAHs coming from vehicle emissions, incomplete biomass combustion, fossil fuels, etc., will be deposited in the top layer of the soil because atmospheric deposition is a common source of soil contamination [24,25,26]. Consequently, the soil is regarded as one of the principal sinks of atmospheric PAHs [27]. Additional mechanisms for the dispersion of PAHs include leaching, volatilization, irreversible combination, plant deposition, and bio-decomposition [28]. Because their hydrophobic properties and chemical stability do not affect bonding to soil particles, PAH concentrations can persist in the soil matrix for a long period of time [29]. As a result, soil can be a useful indicator of PAH pollution and environmental risk [30]. Over the last few decades, soil pollution caused by vehicular emissions has become a serious concern for researchers in southern Asian countries [31,32,33] along with the study of particulate and other gaseous pollution. Heavy metals and other pollutants can be detrimental to human health along with PAHs [34,35,36]. However, very few studies have been conducted on soil PAH contamination as compared to air contamination. As an important industrial area with a high population density, the health risks to the local population from industrial contamination are a serious concern [37,38,39].

The major objective of the present study was to determine the concentration level of the USEPA’s priority PAHs in the soil of industrial areas of two different cities in India, i.e., Jamshedpur in Eastern India and Amravati in central India. The source and the health risk associated with soil PAH exposure were analyzed. The carcinogenic potential of PAHs was described in terms of BaP equivalents. Based on the diagnostic ratios of marker species, the source characteristics of the PAHs were identified, and source contributions were quantified using the diagnostics ratio. There have been few studies on this topic, and the current work could serve as a baseline for future research on the region’s soil PAHs.

## 2. Materials and Method

### 2.1. Study Sites

Our research sites are two Indian industrial cities, i.e., Jamshedpur (Jharkhand) and Amravati (Maharashtra). Jamshedpur is a well-planned industrial city, which is located at 22°80′ N and 86°20′ E. In eastern India, it is a center of an industrial economic zone with significant traffic activity. This city covers an area of 209 km^2^ and is located on the Chota Nagpur Plateau (CNP), which is bordered by a region of lush, green Dalma hills. Jamshedpur has a tropical wet weather and dry climate. This city has a population of about 1.3 million people and a population density of 6400 persons per km^2^, according to the 2011 census (Census 2011). The city is recognized as the largest industrial city in Eastern India due to the presence of significant industrial giants such as TATA Steel formerly known as TISCO, TATA Motors, Tinplate, TATA Timken, Jojobera Cement industry, etc. Other small- to large-scale companies are situated throughout the city which continuously release PAHs into the environment.

Like Jamshedpur, Amravati is also an industrial city, and it is the second largest city in the Vidarbha region after Nagpur. It is the ninth most populated city in Maharashtra, India and is located at 20°93′ N and 77°75′ E with an average elevation of 343 m above sea level. It lies 156 km west of Nagpur and serves as the administrative center of the Amravati district. It has a tropical wet and dry climate with moderate warmth in summers and mild cold weather in winters. The city recorded its highest temperature of 49.1 °C on 25 May 2013 and lowest of 5.0 °C on 9 February 1887. The population density of the city is 3524 inhabitants per km^2^. The total population of the Amravti district is around 2.8 million (Census 2011). The 178.95-hectare-long industrial area of Amravati lies four km from Amravati city, towards Bandera, in Maharashtra, India. Several small- and large-scale businesses operate in and around the city, due to the presence of agglomerates of large and small scale industries.

Additionally, the primary source of emissions into the atmosphere in the surrounding area is the vast network of the highway roadway system near both study sites in Jamshedpur and Amravati. The primary sources of soil, air, and water pollution in this region have been identified as industrial waste emissions, the burning of coal, and vehicle transport emissions.

### 2.2. Sampling

**Figure 1:**
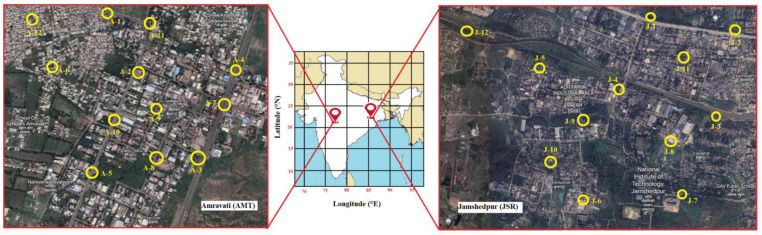
Map of sampling locations of two different industrial cities of India. Amravati and Jamshedpur cities are marked as A1–A12 and J1–J12, respectively.

The soil samples were collected from different locations in the industrial sites in Jamshedpur (JSR) and Amravati (AMT), India, as shown in Figure 1. A total of 12 samples of approx. 100 g were collected from each site of the study. The samples were air-dried at room temperature in the laboratory, and stones, roots, and other debris were removed and sample was well mixed to ensure homogeneity before wrapping with aluminum foil and stored in a cold container to ward off from moisture and air. To prevent contamination, gloves were worn during sampling and the collected samples were sent to the laboratory within 6 h. The moisture was removed from the samples at room temperature for three days before they were crushed and placed in a 1 mm mesh filter. After sieving, the samples were kept at −4 °C until subsequent analysis.

### 2.3. Extraction and Analysis of PAHs from Soil

We measured the 16 PAHs recognized as priority pollutants by the USEPA, i.e., acenaphthylene (Any), acenaphthene (Ane), anthracene (Ant), fluorene (Fle), fluoranthene (Fla), naphthalene (Nap), phenanthrene (Phe), pyrene (Pyr), benzo (a) anthracene (BaA), chrysene (Chr), benzo (b) fluoranthene (BbF), benzo (k) fluoranthene (BkF), benzo (a) pyrene (BaP), indeno (1,2,3-cd) pyrene (IcdP), dibenzo (a,h) anthracene (DahA), and benzo (g, h, i) perylene (BghiP). The total organic carbon (TOC) present in the soil was calculated using the titrimetric method [40,41]. The materials were then extracted with a solvent [42]. The samples were obtained using dichloromethane (DCM) as the solvent in a Soxhlet extractor for 24 h. The solvent was evaporated by a rotary evaporator (Model No-Hei-VAP Core with finger uplift and G1 transverse glassware); afterwards, the solvent fractions were exchanged using 2 mL of n-hexane. The moisture contained in the samples was trapped and cleaned using a silica gel column (3 cm diameter and 30 cm long glass column filled with 10 g of activated silica gel) dissolved in DCM solvent and 5 g anhydrous Na_2_SO_4_. For the recovery investigations, a PAH standard comprised of deuterium-labelled PAH (i.e., Naphthalene-D8, Phenanthrene-D10, Anthracene-D10, and Chrysene-D12) was spiked into 5 g of soil at a concentration of 200 gL^−1^. The final cleaned fraction was then eluted with a mixture of 20 mL n-hexane and DCM (1:1 *v*/*v*). The extracted solvent was concentrated to 20 µL under a gentle flow of pure nitrogen. A standard solution of hexamethyl benzene was introduced (5 µL) before analysis to quantify all analytes. A Gas Chromatograph—Flame ionization detector (GC-FID, model-Agilent 7890B) fitted with an HP-5 MS capillary column (with dimensions of 30 m 0.32 mm 0.25 m) was used to analyze the USEPA’s 16 priority PAHs. A volume of 1 µL from each sample was injected in a split-less fashion. Ultrapure N_2_ gas was used as a transporter gas with a flow of 1.83 mL min^−1^. Initially, the temperature of the oven was set at 70 °C for 1 min which was boosted to 300 °C with at a rate of 5 °C min^−1^ for 20 min. During the respective temperatures of 290 °C and 320 °C, the injector and transmission guide were regulated.

### 2.4. Quality Assurance

To maintain clean and uncontaminated values, the entire glassware setup was rinsed with n-hexane (CAS Number-110/54/3) and deionized water before sampling. Merck and JT Baker supplied the reagents and compounds used in this study (HPLC-grade solvents). Standard solutions of all mentioned PAHs in acetonitrile (ID-3697900), hexamethyl benzene (CAS Number-87/85/4, Manufacturer-Sigma Aldrich, USA), and deuterium-labeled PAHs (HPLC grade solvents) were used. The procedure for routine samples, replicate samples, and surrogate standards [Phenanthrene-D10 (CAS Number-1517/22/2), in the range of 1–15 µg/L] was created from the standard solution by serial dilutions with acetonitrile (JT Baker of HPLC standards) and a calibration curve was drawn for 9 points. To ensure the stability of the instrument, all the standard solutions comprising 16 PAHs must generate a calibration curve for 9 different concentrations. 

The linearity of the curve values were within the acceptable bounds of r^2^ ≥ 0.990 and had a range of 0.9932 to 0.9980 [43]. The deuterated PAHs recovered from the soil samples were found to be around 61 ± 12% for Naphthalene-D8, 82 ± 11% for Phenanthrene-D10, 74% for Anthracene-D10, and 81 ± 12% for Chrysene-D12. The recoveries of standard PAHs were 85–95% for the analyzed sample and blank sample. The blank samples were collected and examined in the laboratory in a similar manner as the samples. The target PAH exposure limits (MDLs) were calculated as three times the concentrations of targeted compounds in spiked blank samples. The limit of quantifications (LOQs) was evaluated as 10 times the standard detection limit (DL). The detection limit for target PAHs was set a standard deviation (SD) plus three times the average concentrations of target complexes in blank samples. The targeted PAHs had detection limits (DL) of 1 ng/g for soil samples, and standards of concentration below those limits were regarded as below detection limits (DLs). The frequency of sample analysis was generally based on the sample collection, generally one sample per day. 

## 3. Heath Risk Assessment

The health risk for a particular PAH can be expressed in terms of toxicity equivalent to BaP (BaP_TEQ_). The BaP_TEQ_ can be calculated as

BaP_TEQ_ = ∑C_i_ × TEF_i_ (1)

where C_i_ is the individual concentration of the PAH (µg/m^3^) and TEF_i_ represents the toxicity equivalent factor. 

Furthermore, the higher possibility of carcinogenic diseases due to exposure to multiple PAHs was estimated in terms of incremental lifetime cancer risk (ILCR) by following the risk assessment guidelines of USEPA [44,45]. The following mathematical expression was used to determine the ILCR:

(2) $${\mathrm{ILCR}}_{\mathrm{injestion}}=\frac{Cs\times \left({CSF}_{injestion}\times \sqrt[{3}]{\frac{BW}{70}}\right)\times {IR}_{Injestion}\times EF\times ED\times CF}{BW\times AT}$$

(3) $${\mathrm{ILCR}}_{\mathrm{dermal}}=\frac{Cs\times \left({CSF}_{dermal}\times \sqrt[{3}]{\frac{BW}{70}}\right)\times {IR}_{dermal}\times SA\times AF\times ABS\times EF\times ED\times CF}{BW\times AT}$$

(4) $${\mathrm{ILCR}}_{\mathrm{inhalation}}=\frac{Cs\times \left({CSF}_{inhalation}\times \sqrt[{3}]{\frac{BW}{70}}\right)\times {IR}_{Inhalation}\times EF\times ED}{BW\times AT\times PEE}$$

where Cs is the sum of the converted concentration of the PAHs based on the TEQ value, CSF represents the carcinogenic slope factor, whose value is 7.30 for ingestion, 25 for dermal absorption, and 3.85 for inhalation [46,47]. 

BW represents the average body weight, 

IR represents the inhalation rate in terms of ingestion, dermal absorption, and inhalation, 

EF is the exposure frequency, 

ED is the exposure duration, 

CF is the conversion factor, 

SA is the dermal exposure area (cm^2^), 

AF is the dermal adherence factor (mg/cm^2^), 

ABS is the dermal adsorption factor, 

PEF is the particle emission factor. 

The detailed parameter values are described in Table 1.

## 4. Result and Discussion

### 4.1. Concentration of PAHs in the Soil

**Figure 2:**
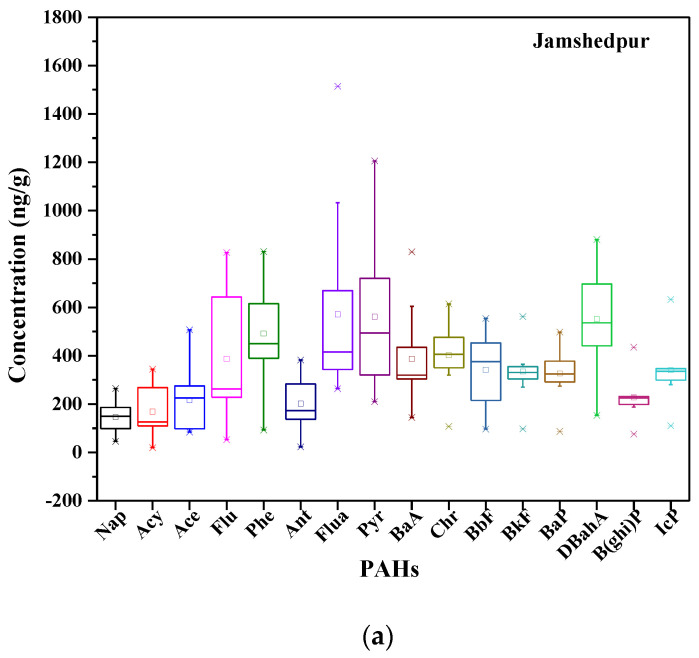

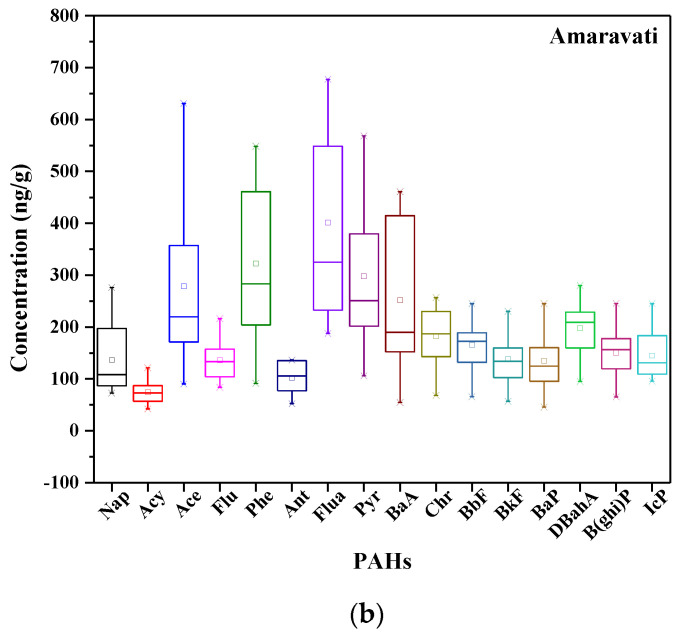
Box plot for the concentration (ng/g) of PAHs in the soil of Jamshedpur and Amaravati industrial sites.

A statistical analysis of the 16 PAHs in the soil samples from two industrial cities in India are presented in Table 2. The total concentration of the 16 PAHs in samples from JSR varied from 1662.90 to 10,879.20 ng/g, whereas the concentration ranged from 1456.22 to 5403.45 ng/g in the soil of AMT. The Σ16PAH concentration in JSR soil was 5655.06 ng/g and 3256.74 ng/g in AMT. The highest level of PAHs was encountered in commercially industrialized zones of both cities. The details of the concentration of PAHs in the soil of both cities are shown in Figure 2. The results showed that PAH concentrations were higher in JSR soils than in AMT soils. Both cities had high concentrations of 4–5-ring PAHs. Greater amounts of Flua, Pyr, and BaA compared to other PAHs were found in both cities. Flua had the highest average concentration in JSR soil at 570.74 ± 318.29 ng/g, followed by Pyr at 326.80 ± 97.11 ng/g and 5-ring DBahA at 551.70 ± 181.82 ng/g. It was also noticed that the observed concentration of PAHs was lower in AMT soils compared to JSR. Flur had the highest average concentration of 401.48 ± 196.42 ng/g, followed by Pyr at 143.18 ± 49.49 ng/g and 5-ring DBahA at 207.33 ± 58.75 ng/g. The details of the concentrations of the PAHs in the soils of both cities are shown in Figure 2.

### 4.2. Study Site PAH Concentrations Compared with Worldwide Levels

The growing contamination of PAHs in the soil of the two industrial cities is correlated with earlier reported PAH levels in different soils throughout the world (Table 3). The average concentration of the 16 PAHs observed was lower than those reported earlier in Delhi, India (5524.3 ng/g) [48], Dhanbad, India (3488 ng/g) [49], Lisbon, Portugal (2717 ng/g) [50], Cape Town, South Africa (4080 ng/g) [51], Xi’an, China (1246 ng/g) [52], Kathmandu, Nepal (1172.8 ng/g) [53], Orlando, USA (3227 ng/g), Tampa, USA (4562 ng/g) [46], and Beijing, China (460 ng/g) [54]. Additionally, the average concentrations of the 16 PAHs found in the current study were greater than those reported in an earlier study of Bokaro, India (139.6 ng/g) [55] and the south of Italy (84.85 ng/g) [56]. Meanwhile, a similar range of average concentrations was reported in Shenzhen, China (360 ng/g) [57], Novi Sad, Serbia (363 ng/g) [58], Pokhare, Nepal (273.7 ng/g) [53], and Jamshedpur, India (366.7 ng/g) [55]. The soils were dominated by higher molecular weight PAHs in both cities, exclusively four-ring PAHs, which is comparable to Orlando and Tampa soils in the United States [53], indicating their pyrogenic origins in these cities [59]. Compared to the present study, the concentration of PAH in the soils from JSR sites was higher than those in a previous study and the concentration of PAHs in AMT industrial sites were similar to those of Orlando, USA, and lower than those of Delhi and Dhanbad of India, Cape Town of South Africa, and Tampa of the USA. The details of the worldwide variation in PAHs in different soils are shown in Table 3.

### 4.3. Health Risk Assessment of PAHs

The BaP_TEQ_ value was determined on the basis of TEFs value and showed that the PAHs BaP and DBahA contributed the highest carcinogenicity for both industrial sites. At the Jamshedpur study site, BaP contributed 31.69% of the carcinogenicity and DBahA contributed 52.48% of the carcinogenicity. At the Amaravati study site, BaP and DBaha contributed 33.01% and 48.63% of the carcinogenicity, respectively. The details of the carcinogenicity contributions are presented in Table 4.

The probabilistic risk assessment due to PAH exposure was quantitatively determined in terms of ILCRs. Oral, respiratory, and dermal exposure the major pathways of ingestion, inhalation, dermal absorption, respectively. In this study, health risk was analyzed for people of three different age groups, i.e., children (1–2 years), adolescents (10–14 years), and adults (30–40 years). The ILCR value for the ingestion, dermal, and inhalation exposure at the Jamshedpur study site were 4.79 × 10^−3^, 5.97 × 10^−3^, and 1.37 × 10^−4^ for children, 2.50 × 10^−3^, 6.69 × 10^−3^, and 2.50 × 10^−4^ for adolescents, and 4.46 × 10^−3^, 3.90 × 10^−3^, and 2.35 × 10^−3^ for adults, respectively.

Similarly, the ILCR values at the Amravati site for ingestion, dermal contact, and inhalation were 2.01 × 10^−3^, 2.49 × 10^−3^, and 5.78 × 10^−5^ for children; for the adolescent age group, the ILCR values were 1.12 × 10^−3^, 2.79 × 10^−3^, and 1.04 × 10^−4^ for the ingestion, dermal, and inhalation routes of exposure; and for the adult group, the ILCR values were 1.86 × 10^−3^, 1.63 × 10^−3^, and 1.72 × 10^−4^ for ingestion, dermal, and inhalation routes of exposure, respectively.

The 90% cumulative probability ILCR value for the Jamshedpur industrial site was calculated to be 10.90 × 10^−3^ for children, 7.19 ×10^−3^ for adolescents, and 10.72 × 10^−3^ for adults. Similarly, for the Amaravati study site, the ILCR value was 4.55 × 10^−3^ for children, 4.02 × 10^−3^ for adolescents, and 3.67 × 10^−3^ for adults. The details of the ILCR values are presented in Table 5. On the basis of toxicity range values, the ILCR value was categorized into three different levels: >10^−4^ was consider a high potential risk, 10^−6^–10^−4^ was considered a moderate potential risk, and <10^−6^ was considered as safe [44]. The result of the present study suggested that the ILCR values for exposure to soils exceeded the high potential risk range for both the Jamshedpur and Amravati industrial sites. Therefore, this study suggests that the primary precaution of emission sources must be identified before conducting the study.

### 4.4. Diagnostic Ratio Analysis

The diagnostic ratio approach is a common method for assessing the sources of PAHs in various environmental media [45,47]. The molecular weight profile of the PAHs determines the environmental fate and transport potentials. In this study, the origins of the PAHs were determined by comparing specified pairs of PAH ratios to the same ratios reported in previous studies. The details of the previous ratio values are described in Table 6.

A previous report stated that a petroleum source was indicated by a ratio of Ant/(Ant + Phe) less than 0.1, whereas a combustion source was indicated by a ratio greater than 0.1 [47]. In the current study, the average ratio value was >0.1 which confirmed pyrogenic combustion sources in both the Jamshedpur and Amravati industrial sites. A ratio value of Fla/(Fla + Pyr) <0.4 indicates a petroleum source, a ratio value of 0.4–0.5 indicates liquid fossil fuel sources, and a ratio value > 0.5 indicates a combustion source [69]. In this study, the average ratio value of Fla/(Fla + Pyr) was >0.5 at the Jamshedpur site indicating coal, wood, or grass combustion sources and for the Amravati study site, the average ratio value was <0.5 indicating petroleum sources. A ratio value of BaA/(BaA + Chr) < 0.2 implies a petroleum source and >0.2 indicates a combustion source [70]. In the present study, the ratio value of BaA/(BaA + Chr) was > 0.2 indicating that the combustion sources for both the Amaravati and Jamshedpur industrial study sites. A ratio value of Phe/Ant < 10 indicates pyrogenic sources and <15 indicates petrogenic sources [71,72]. The average ratio value of Phe/Ant was <10 in the present study which indicated pyrogenic sources. According to the literature, a ratio value of Ind/(Ind + Bghip) <0.2 indicates petrogenic sources, 0.2–0.5 indicates the combustion of fossil fuel from vehicles/other machines as the source, and >0.5 points to the burning of grass/coal/wood [73]. In this analysis, the ratio of Ind/(Ind + Bghip) was >0.5 for Jamshedpur indicating grass/coal/wood combustion and 0.2–0.5 for Amaravati indicating fuel combustion from vehicles and crude oil. Essumang et al. [72] reported that a ratio of BaP/(BaP + Chr) < 0.2 suggested a petroleum source, 0.2–0.35 suggested coal/wood/grass combustion, and >0.35 suggested vehicular emissions as the source. In the present study, the average ratio value of BaP/(BaP + Chr) was >0.35 indicating vehicular emissions as the source for both study sites.

The diagnostic ratios of this study concluded that the major source of PAHs in both study sites was petrogenic sources, coal, wood, and grass combustion, and vehicular emissions. The details of the diagnostic ratios are described in Table 6.

## 5. Summary and Conclusions

In this study, 16 PAHs were analyzed in two industrial cities in India in the states of Jharkhand and Maharashtra that showed higher levels of PAHs with 4–5 rings in both cities. Higher concentrations of Flua, Pyr, and BaA were observed in both cities. In JSR, Flua had the greatest average concentration (570.74 ± 318.29 ng/g), followed by Pyr (561.54 ± 323.67 ng/g) and the 5-ring PAH DBahA (551.70 ± 181.82 ng/g). AMT had a lower soil PAH concentration than JSR. With an average concentration of 401.48 ± 196.42 ng/g, Flua had the highest level, followed by Pyr with 315.74 ± 162.23 ng/g and the 5-ring PAH DBahA with 207.33 ± 58.75 ng/g.

Furthermore, health risk was analyzed in terms of ILCR. The cumulative probability ILCR value for the Jamshedpur industrial site was calculated to be 10.90 × 10^−3^ for children, 7.19 × 10^−3^ for adolescents, and 10.72 × 10^−3^ for adults. Similarly, for the Amaravati study site, the ILCR value was 4.55 × 10^−3^ for children, 4.02 × 10^−3^ for adolescents, and 3.67 × 10^−3^ for adults. The findings of the current study indicate that for both industrial sites in Jamshedpur and Amravati, the ILCR values for exposure to soils was above the high potential risk range value.

The conclusions of the diagnostic ratios suggested that the principal sources of PAHs at both study sites were petroleum combustion, coal, wood, and grass combustion, and the vehicular emissions; thus, there is a requirement for emission source precautions at the study sites.

The limitation of this study is the knowledge gaps about the interactions of soils with environmental fate and biotic behavior which can hinder the accurate assessment of PAH behavior and distribution in soil on a seasonal basis.

This study will motivate us to investigate the mechanisms governing the fate and transport of PAHs in soil, including their sorption, desorption, volatilization, and leaching behaviors. Understanding the factors influencing PAH movement in soil can aid in predicting their distribution and potential for off-site migration. This study also motivates us to explore microbial degradation pathways and the effectiveness of bioremediation strategies for PAH-contaminated soils. Research can focus on identifying microorganisms with high PAH-degrading capabilities and optimizing conditions to enhance microbial activity and PAH removal.

This study will help future investigation of interactions between PAHs in soil and climate change factors, such as altered precipitation patterns, temperature fluctuations, and increased frequency of extreme weather events and assessing how these interactions may influence the behavior, fate, and transport of PAHs in soil systems.

These findings will be helpful for the development of effective strategies for communicating PAH-related risks to stakeholders, including communities, policymakers, and industry and engaging in collaborative efforts to bridge the gap between scientific research, policy development, and practical implementation of soil management and remediation strategies.

## Figures and Tables

**Table 1:** The parameter values used for the determination of ILCR for children, adolescents, and adults.

Parameter	Unit	Child	Adolescent	Adult
Body weight (BW)	kg	15	45	62
Ingestion rate (IR ingestion)	mg/d	200	100	100
Exposure frequency (EF)	d/year	350	350	350
Exposure duration (ED)	year	6	14	30
Average lifetime span (AT)	d	26,280	26,280	26,280
Surface area (SA)	cm^2^/d	2800	2800	2800
Dermal surface factor (AF)	mg/cm^2^	0.2	0.2	0.07
Dermal absorption fraction (ABS)	unit less	0.13	0.13	0.13
Inhalation rate (IR inhalation)	m^3^/d	10.9	17.7	17.5
Particulate emission factor	m^3^/kg	1.36 × 10^9^	1.36 × 10^9^	1.36 × 10^9^

**Table 2:** The concentration (ng/g) of individual PAHs in the industrial site soils of Jamshedpur and Amravati.

PAHs	Abbreviation	No. of Rings	Jamshedpur				Amravati			
			Average	SD	Min	Max	Average	SD	Min	Max
Naphthalene	Nap	2	145.77	59.36	46.06	264.52	136.40	66.19	<DL	276.2
Acenaphthylene	Acy	3	168.83	80.22	19.40	344.50	77.63	56.62	50.064	121.4
Acenaphthene	Ace	3	217.08	112.63	83.84	507.11	296.09	157.61	150.696	631.2
Fluorene	Flu	3	386.82	209.00	52.58	826.30	140.62	138.28	91.56	216.3
Phenanthrene	Phe	3	491.76	220.66	93.03	830.80	343.45	141.76	179.34	548.632
Anthracene	Ant	3	201.32	209.27	23.12	382.10	106.79	154.88	<DL	154.8821
Fluoranthene	Flua	4	570.74	318.29	263.62	1514.02	401.48	196.42	<DL	677.306
Pyrene	Pyr	4	561.54	323.67	209.97	1205.90	315.74	162.23	184.212	568.327
Benzo[a]anthracene	BaA	4	387.43	256.21	144.34	829.01	269.61	133.81	139.188	460.762
Chrysene	Chr	4	402.16	144.38	106.90	613.96	192.59	103.85	125.664	256.742
Benzo[b]fluoranthene	BbF	5	341.27	130.72	96.56	554.54	174.70	43.59	116.088	245.2
Benzo[k]fluoranthene	BkF	5	334.65	121.02	97.87	562.11	145.58	45.23	90.048	230.5
Benzo[a]pyrene	BaP	5	326.80	97.11	86.68	497.85	143.18	49.49	83.832	245.2
Dibenzo[ah]anthracene	DBahA	5	551.70	181.82	153.22	880.01	207.33	58.75	140.448	280.3
Benzo[ghi]perylene	B(ghi)P	6	227.54	214.34	75.69	434.69	158.35	47.58	105.084	245.3
Indeno[123-cd]pyrene	IcP	6	339.65	111.22	110.01	631.81	147.19	40.01	<DL	245.2
Σ16PAHs			5655.06	2789.92	1662.90	10,879.20	3256.74	1596.28	1456.22	5403.45

**Table 3:** Descriptive statistics for the concentration (ng/g) of PAHs in the soils of different areas of the world.

Sl No.	No. of PAHs	Location	Min	Max	Mean	Reference
1	15	Izmir, Turkey	11	4628	-	[60]
2	16	Western Canada	2.03	789	-	[61]
3	16	Schwaebische Alb, Germany	-	1140	-	[62]
4	16	Pearl River Delta, China	28	710	-	[63]
5	16	Delhi, India	1550.9	11,460	5524.3	[48]
6	16	London, UK	4000	67,000	-	[64]
7	10	Hanoi, Vietnam	0.34	43.7	-	[65]
8	15	Kumming, China	101.64	693.3	-	[66]
9	13	Dhanbad, India	1019	10,856	3488	[49]
10	16	Shenzhen, China	2	6745	360	[57]
11	16	Lisbon, Portugal	6	73,395	2717	[50]
12	16	Novi, Sad, Serbia	22	2247	363	[58]
13	8	Cape Town, South Africa	<DL	13,880	4080	[51]
14	16	Xi’an, China	149.9	5770	1246	[52]
15	15	Pokhare, Nepal	17.1	1852.5	273.7	[53]
16	15	Kathmandu, Nepal	20.6	6219.3	1172.8	
17	16	Orlando, USA	43	30,428	3227	[46]
18	16	Tampa, USA	59	58,640	4562	
19	16	South of Italy	7.62	755	84.85	[56]
20	7	Delhi, India	<DL	862	-	[67]
21	16	China	6.94	5870	-	[68]
22	16	Japan	31.9	507	-	
23	16	South Korea	6.41	161	-	
24	16	Vietnam	26.9	864	-	
25	16	India	14.3	1590	-	
26	16	Beijing, China	66	6867	460	[54]
27	16	Bokaro, India	<DL	670.6	139.6	[55]
28	16	Jamshedpur, India	38.6	1781.2	366.7	[55]
29	16	Jamshedpur, India	1662.90	10,879.20	5655.06	This Study
30	16	Amravati, India	1456.22	5403.45	3256.74	

**Table 4:** The values of TEF and BaP_TEQ_ for the Jamshedpur and Amaravati study sites.

PAHs	Jamshedpur				Amaravati		
	TEFs	PAHs (ng/g)	BaP_TEQ_	BaP_TEQ_ %	PAHs (ng/g)	BaP_TEQ_	BaP_TEQ_ %
Nap	0.001	156.66	0.16	0.01	124.71	0.12	0.03
Acy	0.001	133.71	0.13	0.01	71.98	0.07	0.02
Ace	0.001	265.44	0.27	0.03	269.83	0.27	0.07
Flu	0.001	310.42	0.31	0.03	130.50	0.13	0.03
Phe	0.001	440.93	0.44	0.04	279.53	0.28	0.07
Ant	0.01	162.49	1.62	0.15	100.68	1.01	0.26
Flua	0.001	752.54	0.75	0.07	340.46	0.34	0.09
Pyr	0.001	705.15	0.71	0.07	288.07	0.29	0.07
BaA	0.1	458.95	45.89	4.36	223.87	22.39	5.73
Chr	0.01	408.27	4.08	0.39	170.58	1.71	0.44
BbF	0.1	376.01	37.60	3.57	159.66	15.97	4.09
BkF	0.1	357.23	35.72	3.39	131.90	13.19	3.38
BaP	1	333.67	333.67	31.69	128.92	128.92	33.01
DBahA	1	552.66	552.66	52.48	189.89	189.89	48.63
B(ghi)P	0.01	246.17	2.46	0.23	149.19	1.49	0.38
IcP	0.1	365.87	36.59	3.47	144.30	14.43	3.70
Total			1029.66	100.00		430.51	100.00

**Table 5:** The total ILCR values for children, adolescents, and adults at the Jamshedpur and Amaravati industrial sites.

	Jamshedpur			Amravati		
	Children	Adolescents	Adults	Children	Adolescents	Adults
ILCR	4.79 × 10^−3^	2.50 × 10^−4^	4.46× 10^−3^	2.01 × 10^−3^	1.12 × 10^−3^	1.86 × 10^−3^
	5.97 × 10^−3^	6.69 × 10^−3^	3.90 × 10^−3^	2.49 × 10^−3^	2.79 × 10^−3^	1.63 × 10^−3^
	1.37 × 10^−4^	2.50 × 10^−4^	2.35 × 10^−3^	5.78 × 10^−5^	1.04 × 10^−4^	1.72 × 10^−4^

**Table 6:** Diagnostic ratio analysis for the PAH source assignment.

PAHs	Range	Sources	Jamshedpur	Amaravati	Reference
Ant/(Ant + Phe)	<0.1 >0.1	Petrogenic Pyrogenic combustion	0.28	0.22	[47]
Flua/(Flua + Pyr)	<0.5 >0.5	Petrogenic Coal, wood or grass combustion	0.50	0.45	[69]
BaA/(BaA + Chr)	<0.2 >0.2	Petrogenic Pyrogenic/combustion	0.48	0.55	[70]
Phe/Ant	<10 <15	Pyrogenic Petrogenic	2.69	2.89	[71]
Ind/(Ind + Bghip)	<0.2 0.2–0.5 >0.5	Petrogenic Fuel combustion (vehicles and crude oil) Grass/coal/wood combustion	0.60	0.45	[72]
BaP/(BaP + Chr)	<0.2 0.2–0.35 >0.35	Petroleum Coal, wood, or grass combustion Vehicular combustion	0.45	0.42	[73]

## Data Availability

The data can be available from the first or corresponding author on requirement and upon official request.

## References

[1] IARC (1983). Polynuclear Aromatic Compounds, Part 1, Chemical, Environmental and Experimental Data. IARC Monographs on the Evaluation of the Carcinogenicity Risk of Chemical to Humans 32.

[2] Boeuf B., Fritsch O., Martin-Ortega J. (2016). Undermining European environmental policy goals? The EU water framework directive and the politics of exceptions. Water.

[3] Yang F., Zhang Q., Guo H., Zhang S. (2010). Evaluation of cytotoxicity, genotoxicity and teratogenicity of marine sediments from Qingdao coastal areas using in vitro fish cell assay, comet assay and zebrafish embryo test. Toxicol. In Vitro.

[4] Kumar A., Sankar T.K., Sethi S.S., Ambade B. (2020). Characteristics, Toxicity, Source identification and Seasonal variation of Atmospheric Polycyclic Aromatic Hydrocarbons over East India. Environ. Sci. Pollut. Res..

[5] Peng C., Chen W., Liao X., Wang M., Ouyang Z., Jiao W., Bai Z. (2012). Vegetative cover and PAHs accumulation in soils of urban green space. Environ. Pollut..

[6] (2003). Australian Protection Agency Report. http://www.scew.gov.au/archive/air/pubs/atem/at_rev_pahs_health_review_200305.pdf.

[7] Harrison R.M., Smith D.J.T., Luhana L. (1996). Source apportionment of atmospheric polycyclic aromatic hydrocarbons collected from an urban location in Birmingham, U.K. Environ. Sci. Technol..

[8] Marr L.C.W., Harley R.A., Miguel A.H. (1999). Characterization of polycyclic aromatic hydrocarbons in motor vehicle fuels and exhaust emission. Environ. Sci. Technol..

[9] Wang X.H., Ye C.X., Yin H.L., Zhuang M.Z., Wu S.P., Mu J.L., Hong H.S. (2007). Contamination of polycyclic aromatic hydrocarbons bound to PM10/PM2. 5 in Xiamen, China. Aerosol Air Qual. Res..

[10] Mostert M.M.R., Ayoko G.A., Kokot S. (2010). Application of chemometrics to analysis of soil pollutants. TrAC Trends Anal. Chem..

[11] Bull K. (2003). Protocol to the 1979 Convention on Long-Range Trans Boundary Air Pollution on Persistent Organic Pollutants: The 1998 Agreement for the UNECE Region.

[12] Liu Y., Liu L., Lin J.M., Tang N., Hayakawa K. (2006). Distribution and characterization of polycyclic aromatic hydrocarbon compounds in airborne particulates of East Asia, China. Particuology.

[13] Shibamoto T. (1998). Chromatographic Analysis of Environmental and Food Toxicants.

[14] U.S. Environmental Protection Agency (USEPA) (1986). Guidelines for Carcinogen Risk Assessment—Federal Register, 51(185), 33992–34003 EPA/630/R-00/004.

[15] Ravindra K., Sokhi R., Van Grieken R. (2008). Atmospheric polycyclic aromatic hydrocarbons: Source attribution, emission factors and regulation. Atmos. Environ..

[16] (2012). United States Environmental Protection Agency (USEPA), Washington, DC, USA. http://www.epa.gov/reg3hwmd/risk/.

[17] Menzie C.A., Potocki B.B., Santodonato J. (1992). Exposure to carcinogenic PAHs in environment. Environ. Sci. Technol..

[18] Crimmins B., Dickerson R., Doddridge B., Baker J. (2004). Particulate polycyclic aromatic hydrocarbons in the Atlantic and Indian Ocean atmospheres during the Indian Ocean Experiment and Aerosols 99: Continental sources to the marine atmosphere. J. Geophys. Res. Atmos. JGR.

[19] Ding X., Wang X., Xie Z., Xiang C., Mai B., Sun L., Zheng M., Sheng G., Fu J., Pöschl U. (2007). Atmospheric polycyclic aromatic hydrocarbons observed over the North Pacific Ocean and the Arctic area: Spatial distribution and source identification. Atmos. Environ..

[20] Friedman C.L., Zhang Y., Selin N.E. (2013). Climate change and emissions impacts on atmospheric PAH transport to the Arctic. Environ. Sci. Technol..

[21] Wania F., Mackay D. (1993). Global fractionation and cold condensation of low volatility organochlorine compounds in polar-regions. Ambio.

[22] Wania F., Mackay D., Li Y.F., Bidleman T.F., Strand A. (1999). Global chemical fate of alpha-hexachlorocyclohexane. 1. Evaluation of a global distribution model. Environ. Toxicol. Chem..

[23] Agarwal T. (2009). Concentration level, pattern and toxic potential of PAHs in traffic soil of Delhi. India J. Hazard. Mater..

[24] ATSDR (1995). Toxicological Profile for Polycyclic Aromatic Hydrocarbons.

[25] Wang X.L., Tao S., Dawson R., Cao J., Li B.G. (2002). Modeling the transfer and the fate of benzo(a)pyrene in wastewater irrigated area in Tianjin. J. Environ. Qual..

[26] Tao S., Cao H.Y., Liu W.X., Li B.G., Cao J., Xu F.L., Wang X.J., Conveny J.R., Shen W.R., Qing B.P. (2003). Fate modeling of phenanthrene with regional variation in Tianjin. China Environ. Sci. Technol..

[27] Morillo E., Romero A.S., Madrid L., Villaverde J., Maqueda C. (2008). Characterization and sources of PAHs and potentially toxic metals in urban environments of Sevilla (Southern Spain). Water Air Soil Pollut..

[28] Reilley K.A., Banks M.K., Schwab A.P. (1996). Dissipation of polycyclic aromatic hydrocarbons in the rhizosphere. J. Environ. Qual..

[29] Wilcke W., Amelung W., Martius C., Garcia M.V.B., Zech W. (2000). Biological sources of polycyclic aromatic hydrocarbons (PAHs) in the Amazonian rain forest. J. Plant Nutr. Soil Sci..

[30] Liang J., Guangjun M., Hailan F., Liang C., Peter C. (2011). Polycyclic aromatichydrocarbon concentration representing different land use categories in Shanghai. Environ. Earth Sci..

[31] Ray S., Khillare P.S., Agarwal T., Shridhar V. (2008). Assessment of PAHs in soil around the international airport in Delhi, India. J. Hazard. Mater..

[32] Agarwal T., Khillare P.S., Shridhar V. (2006). PAHs contamination in the bank sediment of the Yamuna River, Delhi, India. Environ. Monit. Assess..

[33] Sharma H., Jain V.K., Khan Z.H. (2007). Characterization and source identification of polycyclic aromatic hydrocarbons (PAHs) in the urban environment of Delhi. Chemosphere.

[34] Golia E.E., Papadimou S.G., Cavalaris C., Tsiropoulos N.G. (2021). Level of Contamination Assessment of Potentially Toxic Elements in the Urban Soils of Volos City (Central Greece). Sustainability.

[35] Gu Y.-G., Gao Y.-P., Lin Q. (2016). Contamination, bioaccessibility and human health risk of heavy metals in exposed-lawn soils from 28 urban parks in southern China’s largest city, Guangzhou. Appl. Geochem..

[36] Wijesiri B., Egodwatta P., McGree J., Goonetilleke A. (2015). Process variability of pollutant build-up on urban road surfaces. Sci. Total Environ..

[37] Behera S.N., Sharma M., Shukla S.P. (2015). Characterization of gaseous pollutants and water-soluble inorganic ions in PM2.5 during summer-time at an urban site of north India. J. Hazard. Toxic Radioact. Waste.

[38] Singh D., Shukla S.P., Sharma M., Behera S.N., Mohan D., Singh N.B., Pandey G. (2014). GIS based on-road vehicular emission inventory for Lucknow City. J. Hazard. Toxic Radioact. Waste.

[39] Behera S.N., Sharma M., Nayak P., Shukla S.P., Gargava P. (2014). An approach for evaluation of proposed air pollution control strategy to reduce levels of nitrogen oxides in an urban environment. J. Environ. Plan. Manag..

[40] Jackson M.L. (1973). Soil Chemical Analysis.

[41] Walkley A., Black C.A. (1934). An estimation method for determination of soil organic matter and a proposed modification of the chromic acid titration method. Soil Sci..

[42] Devi N.L., Yadav I.C., Shihua Q., Dan Y., Zhang G., Raha P. (2016). Environmental carcinogenic polycyclic aromatic hydrocarbons in soil from Himalayas, India: Implications for spatial distribution, sources apportionment and risk assessment. Chemosphere.

[43] Adeniji A.O., Okoh O.O., Okoh A.I. (2017). Petroleum hydrocarbon fingerprints of water and sediment samples of Buffalo River Estuary in the Eastern Cape Province, South Africa. J. Anal. Methods Chem..

[44] Chen S.C., Liao C.M. (2006). Health risk assessment on human exposed to environmental polycyclic aromatic hydrocarbons pollution sources. Sci. Total Environ..

[45] Jiao H., Wang Q., Zhao N., Jin B., Zhuang X., Bai Z. (2017). Distributions and sources of polycyclic aromatic hydrocarbons (PAHs) in soils around a chemical plant in shanxi, China. Int. J. Environ. Res. Public Health.

[46] Liu Y.G., Gao P., Su J., Da Silva E.B., de Oliveira L.M., Townsend T., Xiang P., Ma L. (2019). PAHs in urban soils of two Florida cities: Background concentrations, distribution, and sources. Chemosphere.

[47] Bucheli T.D., Blum F., Desaules A., Gustafsson Ö. (2004). Polycyclic aromatic hydrocarbons, black carbon, and molecular markers in soils of Switzerland. Chemosphere.

[48] Singh D.P., Gadi R., Mandal T.K. (2012). Levels, sources, and toxic potential of polycyclic aromatic hydrocarbons in urban soil of Delhi, India. Hum. Ecol. Risk Assess. Int. J..

[49] Suman S., Sinha A., Tarafdar A. (2016). Polycyclic aromatic hydrocarbons (PAHs) concentration levels, pattern, source identification and soil toxicity assessment in urban traffic soil of Dhanbad, India. Sci. Total Environ..

[50] Marinho R.A.P., Shepherd T., Nowell G., Cachada A., Duarte A.C., Cave M., Wragg J., Patinha C., Dias A., Rocha F. (2016). Source and pathway analysis of lead and polycyclic aromatic hydrocarbons in Lisbon urban soils. Sci. Total Environ..

[51] Omores R.A., Wewers F., Ikhide P.O., Farrar T., Giwa A. (2017). Spatio-temporal distribution of polycyclic aromatic hydrocarbons in urban soils in Cape Town, South Africa. Int. J. Environ. Res..

[52] Bao H., Hou S., Niu H., Tian K., Liu X., Wu F. (2018). Status, sources, and risk assessment of polycyclic aromatic hydrocarbons in urban soils of Xi’an, China. Environ. Sci. Pollut. Res..

[53] Pokhrel B., Gong P., Wang X., Chen M., Gao S. (2018). Distribution, sources, and air-soil exchange of OCPs, PCBs and PAHs in urban soils of Nepal. Chemosphere.

[54] Qu Y., Gong Y., Ma J., Wei H., Liu Q., Liu L., Wu H., Yang S., Chen Y. (2020). Potential sources, influencing factors, and health risks of polycyclic aromatic hydrocarbons (PAHs) in the surface soil of urban parks in Beijing, China. Environ. Pollut..

[55] Ambade B., Sethi S.S., Chintalacheruvu M.R. (2023). Distribution, risk assessment, and source apportionment of polycyclic aromatic hydrocarbons (PAHs) using positive matrix factorization (PMF) in urban soils of East India. Environ. Geochem. Health.

[56] Thiombane M., Albanese S., Di Bonito M.D., Lima A., Zuzolo D., Rolandi R., Qi S., De Vivo B. (2019). Source patterns and contamination level of polycyclic aromatic hydrocarbons (PAHs) in urban and rural areas of Southern Italian soils. Environ. Geochem. Health.

[57] Zhang D., Wang J., Zeng H. (2016). Soil polycyclic aromatic hydrocarbons across urban density zones in Shenzhen, China: Occurrences, source apportionments, and spatial risk assessment. Pedosphere.

[58] Skrbic B.D., Ðurišić-Mladenović N., Tadić Ð.J., Cvejanov J.Ð. (2017). Polycyclic aromatic hydrocarbons in urban soil of Novi Sad, Serbia: Occurrence and cancer risk assessment. Environ. Sci. Pollut. Res..

[59] Chen Y., Tian C., Li K., Cui X., Wu Y., Xia Y. (2016). Influence of thermal maturity on Carbon isotopic composition of individual aromatic hydrocarbons during anhydrous closed-system pyrolysis. Fuel.

[60] Bozlaker A., Muezzinoglu A., Odabasi M. (2008). Atmospheric concentrations, dry deposition and air–soil exchange of polycyclic aromatic hydrocarbons (PAHs) in an industrial region in Turkey. J. Hazard. Mater..

[61] Choi S.-D., Shunthirasingham C., Daly G.L., Xiao H., Lei Y.D., Wania F. (2009). Levels of polycyclic aromatic hydrocarbons in Canadian mountain air and soil are controlled by proximity to roads. Environ. Pollut..

[62] Schwarz K. (2010). Atmogenic Pollutants as Reactive Tracers for Identification and Quantification of Important Transport Processes in a Karst Area at the Catchment Scale. Ph.D. Thesis.

[63] Liu G., Yu L., Li J., Liu X., Zhang G. (2011). PAHs in soils and estimated air–soil exchange in the Pearl River Delta, south China. Environ. Monit. Assess..

[64] Vane C.H., Kim A.W., Beriro D.J., Cave M.R., Knights K., Moss-Hayes V., Nathanail P.C. (2014). Polycyclic aromatic hydrocarbons (PAH) and polychlorinated biphenyls (PCB) in urban soils of Greater London, UK. Appl. Geochem..

[65] Pham C.T., Tang N., Toriba A., Hayakawa K. (2015). Polycyclic aromatic hydrocarbons and nitro-polycyclic aromatic hydrocarbons in atmospheric particles and soil at a traffic site in Hanoi, Vietnam. Polycycl. Aromat. Compd..

[66] Yang X., Ren D., Sun W., Li X., Huang B., Chen R., Lin C., Pan X. (2015). Polycyclic aromatic hydrocarbons associated with total suspended particles and surface soils in Kunming, China: Distribution, possible sources, and cancer risks. Environ. Sci. Pollut. Res..

[67] Gupta H., Kumar R. (2019). Distribution of some polycyclic aromatic hydrocarbons in urban soils of Delhi, India. Environ. Technol. Innov..

[68] Hong W.J., Li Y.F., Li W.L., Jia H., Minh N.H., Sinha R.K., Moon H.B., Nakata H., Chi K.H., Kannan K. (2020). Soil concentrations and soil-air exchange of polycyclic aromatic hydrocarbons in five Asian countries. Sci. Total Environ..

[69] Yunker M.B., Macdonald R.W., Vingarzan R., Mitchell R.H., Goyette D., Sylvestre S. (2002). PAHs in the Fraser River basin: A critical appraisal of PAH ratios as indicators of PAH source and composition. Org. Geochem..

[70] Christensen E.R., Bzdusek P.A. (2005). PAHs in sediments of the Black River and the Ashtabula River, Ohio: Source apportionment by factor analysis. Water Res..

[71] Maliszewska-Kordybach B., Smreczak B., Klimkowicz-Pawlas A., Terelak H. (2008). Monitoring of the total content of polycyclic aromatic hydrocarbons (PAHs) in arable soils in Poland. Chemosphere.

[72] Essumang D.K., Kowalski K., Sogaard E.G. (2011). Levels, distribution and source characterization of polycyclic aromatic hydrocarbons (PAHs) in top soils and roadside soils in Esbjerg, Denmark. Bull. Environ. Contam. Toxicol..

[73] Mannino M.R., Orecchio S. (2008). Polycyclic aromatic hydrocarbons (PAHs) in indoor dust matter of Palermo (Italy) area: Extraction, GC–MS analysis, distribution and sources. Atmos. Environ..

